# The race against time

**DOI:** 10.2471/BLT.15.020115

**Published:** 2015-01-01

## Abstract

Two promising vaccines for Ebola virus disease are being tested in record time and – if all goes well – could be used to stop the outbreak ravaging parts of western Africa. Fiona Fleck and Ana Lesher report.

The first time Dr Ripley Ballou, Vice President of GlaxoSmithKline (GSK) Biologicals, contacted the World Health Organization (WHO) about a promising Ebola vaccine candidate, it was 24 March 2014 – the day WHO issued news of the Ebola virus disease outbreak in Guinea.

“I was told that since there were no human data, there were no policies or pathways for its use in the current outbreak,” Ballou recalls. ”There was also a strong belief that the usual approach of containments would stop the outbreak.”

When WHO called Ballou a few months later the picture had changed, and on 8 August WHO declared the outbreak a public health emergency of international concern.

“We realized this outbreak was different and the approach used successfully in previous outbreaks – detecting and isolating cases, identifying contacts and safely burying the deceased – was not working,” says Dr Marie-Paule Kieny, WHO Assistant Director-General for Health Systems and Innovation.

Within a month, Kieny and her team hosted a gathering of more than 200 of the world’s leading vaccine experts from industry, academia and regulatory authorities as well as public health officials from the countries affected and experts in filoviruses and viral haemorrhagic diseases.

The 4‒5 September meeting reviewed the pipeline of therapies and vaccines for Ebola and whether it was feasible to test them in time to help stop the outbreak in the worst affected countries: Guinea, Liberia and Sierra Leone. 

“We identified two promising vaccine candidates which we agreed should be tested as quickly as possible without compromising safety and scientific rigour,” says Kieny, a microbiologist, who worked in vaccine and cancer research in France for 20 years before joining WHO in 2001 to establish the WHO Initiative for Vaccine Research. 

Since then, Kieny has overseen the development and licensing of new vaccines for meningitis and pandemic influenza in developing countries, by pioneering the transfer of technology and know-how. Now, she is devoting much of her time to galvanizing international collaborations to speed up the pipeline for Ebola vaccines and therapies.

“Usually it takes five to 10 years to research and develop a new vaccine – we foresee doing this within a year or 18 months – which is unprecedented,” Kieny says.

For Ballou, there is no question of cutting corners. “We’re pressing the system as hard as we can. Things that take 2 or 3 months, are getting done in a fraction of that time.”

“Things that take 2 or 3 months, are getting done in a fraction of that time.”

Marie-Paule Kieny

One of the vaccine candidates is a recombinant chimpanzee adenovirus expressing an Ebola virus protein or ChAd3, that was developed by GSK and the US National Institute of Allergy and Infectious Diseases (NIAID). In one study, researchers found that a single dose of ChAd3 protected all 16 animals given a lethal dose of the Ebola virus. 

The other vaccine – a recombinant vesicular stomatitis virus (rVSV) expressing an Ebola virus protein, developed by the Public Health Agency of Canada and US-based NewLink Pharmaceuticals – protected all 20 animals given a lethal dose of the virus. Newlink recently licensed the technology to Merck, which is taking responsibility for the future development of the vaccine.

A third vaccine developed by Johnson & Johnson is due to be tested in Phase 1 clinical trials starting this month. In addition, five US-based companies, Profectus Biosciences, Protein Sciences, Novavax, Vaxart and Inovio, are each developing their own vaccine candidates, while three further candidates are being developed in the Russian Federation and other European countries.

**Figure:**
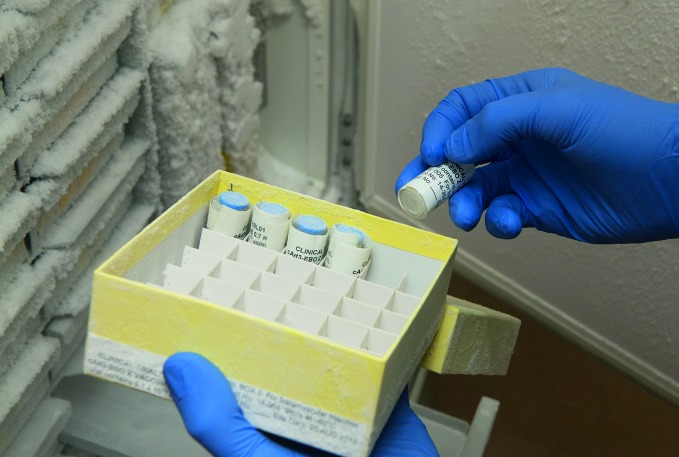
Supplies of vaccine for the Oxford-based clinical trial are kept in a biosafety level 4 laboratory.

University of Oxford

Phase 1 trials to test the safety of the GSK–NIAID vaccine started in the United Kingdom and United States in September and continued in Mali and Switzerland in October – aiming to enrol 260 participants.

Phase 1 trials of rVSV started in the United States in October and Gabon, Germany, Kenya and Switzerland in November with 250 participants. Initial data from the trials of the two vaccines became available in December. 

First results from a small phase 1 trial in the USA showed that the GSK-NIAID vaccine was well-tolerated and produced an immunological response in each of the 20 healthy adult volunteers who received it. 

If further safety data from trials in Europe and Africa are positive too, Phase II trials in non-affected African countries and Phase III in Guinea, Liberia and Sierra Leone will start this month. Data on whether the vaccines protect people from Ebola virus disease infection may be available as early as April, which may be enough evidence for mass vaccination.

When Liberian infectious diseases expert, Dr Stephen B Kennedy, was appointed by the government as coordinator for research of the incident management systems, to oversee all Ebola-related research in his country, it was the challenge of his life. 

“Our hospitals and other health facilities were unable to cope with the epidemic, our laboratories were inadequate with reduced capacity because many Liberians have left the country. Despite that, we managed to recruit a team of 12 competent Liberian researchers, immunologists, physicians, ethicists and communications experts.”

In 2007, Kennedy had helped to establish an infectious diseases research centre in Liberia – the country that has seen the most Ebola cases and deaths in the current outbreak – “so prior to Ebola we had built up some research infrastructure”.

“Prior to Ebola we had built up some research infrastructure.”

Stephen B Kennedy

Kennedy and his team aim to recruit about 27 000 healthy adults for the Phase III efficacy trial of the two vaccines (rVSV and ChAd3) in double-blinded randomized controlled trials (RCT). RTCs are considered the gold standard in terms of producing the strongest evidence, since a group that has received the vaccine will be compared with a group that has not.

“Suitable study sites are being identified and prepared, management and operational systems formulated, regulatory and ethical issues assessed, and communication and community mobilization strategies planned in preparation for the phase III trial,” Kennedy says, adding that he also plans a phase II safety and immunologic response study of about 600 healthy adults to assess the country’s capacity to effectively conduct the large study. 

In Guinea, clinical trials are being coordinated by Professor Mandy Kader Konde, a retired WHO expert on emerging infections, who worked on outbreaks of Ebola in 1995 and Marburg virus in 1998 in the Democratic Republic of the Congo. Trials in Sierra Leone are being coordinated by Dr Samuel A Kargbo, the Director of Reproductive and Child Health in the country’s Ministry of Health and Sanitation. 

Education and preparation of participants is vital for them to understand why some will receive the vaccine for Ebola while others won’t. “We don’t know whether the vaccine works, and until we know that, there is no ethical issue about not providing it to some people. More importantly, an RCT is the fastest, most reliable way to find out whether it actually works,” Ballou says. 

Dr Cheikh Niang, a medical anthropologist from Senegal, who was commissioned by WHO to prepare the first socio-anthropological study of the effect of Ebola virus disease on communities in Sierra Leone and is currently working on outbreak patterns in Mali, agrees. 

“Strong communication efforts need to be done before, during and after any trial. Local communities can accept the goals and the rationale of trials if they are well informed,” Niang says. 

While the two vaccine producers have enough doses for the trials, if early positive results suggest that either vaccine should be rolled out in regions affected by the outbreak in West Africa, there may not be enough vaccine supplies until mid-2015. 

In November, the GAVI Alliance, which funds vaccines in 73 developing countries, hosted a meeting with donors, public health experts and scientists, who called for immediate “large-scale vaccine use planning … given the epidemiologic unknowns in the current epidemic and potential risk of further spread,” according to the meeting report. 

Modelling studies show that even if part of the population in the three countries is vaccinated against the virus, herd immunity would increase considerably. For that reason, it may only be necessary to target health-care workers, carry out ring vaccinations around clusters of cases or target certain geographical areas. 

Despite that, planners should reckon on having an estimated 12–20 million vaccine doses in the three countries. For even if less is needed, excess vaccine may be required in other countries and can be stockpiled for future outbreaks.

**Figure:**
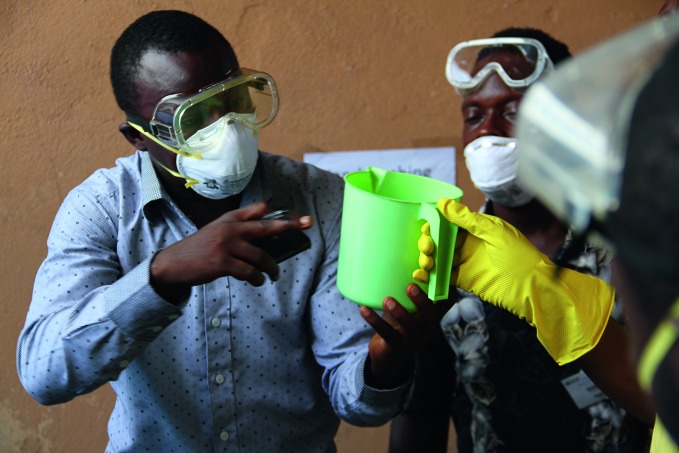
Health workers training to care for patients with Ebola virus disease. If clinical trials go ahead this month in Liberia, health workers such as these will be among the participants.

WHO/Peter Desloovere

 “Hopefully, we will not only come up with a vaccine in time for use in the current outbreak but also one that can be deployed in future Ebola emergencies,” says Kieny. 

The US government started developing what became the GSK vaccine in the 1990s, in response to bioterror threats and fears that the Ebola virus had been weaponized. But progress has been slow for such vaccines for several reasons. The market for vaccines needed mainly in low-income countries is not considered lucrative. Clinical trials needed to license such products have been difficult, given that previous outbreaks were too short, small and self-contained for conducting such trials. There was also less demand for these vaccines, since previous outbreaks were halted by infection control measures.

If and when either of the two vaccines proves to be safe and effective, how will a sufficient supply of doses to help quell the outbreak be financed? “We are looking closely at how GAVI could use traditional or innovative finance mechanisms to support the scale-up of production and procurement of an approved Ebola vaccine,” says Dr Seth Berkley, chief executive of GAVI, speaking ahead of the GAVI board meeting last month. As of 3 December, there had been 17 145 reported cases of Ebola virus disease, with 6070 reported deaths.

“As well as supporting large-scale vaccination campaigns if required, we are also assessing the options for stockpiling a vaccine for future outbreaks,” Berkley says adding: “Clearly whatever happens, tackling Ebola will require funding efforts from many sources.” 

